# RIG-I Stimulation Enhances the Effector Function and Proliferation of Primary Human CD8+ T Cells

**DOI:** 10.3390/ijms27073058

**Published:** 2026-03-27

**Authors:** Adham Abuelola Mohamed, Christina Wallerath, Charlotte Hunkler, Gunther Hartmann, Sanda Stankovic, Andrew G. Brooks, Martin Schlee

**Affiliations:** 1 Department of Clinical Chemistry and Clinical Pharmacology, University Hospital Bonn, 53127 Bonn, Germany; christina.wallerath@ukbonn.de (C.W.); charlotte.hunkler@ukbonn.de (C.H.); gunther.hartmann@uni-bonn.de (G.H.); 2 Department of Microbiology and Immunology, The Peter Doherty Institute for Infection and Immunity, University of Melbourne, Melbourne, VIC 3010, Australiaagbrooks@unimelb.edu.au (A.G.B.); 3 Department of Internal Medicine II, Hematology, Oncology, Clinical Immunology and Rheumatology, University Hospital Tübingen, University of Tübingen, 72076 Tübingen, Germany

**Keywords:** CD8 T cells, influenza A virus, nucleic acid sensing, RIG-I, IFN-I

## Abstract

Cytotoxic CD8 T lymphocytes are crucial in antiviral immune responses. However, their recruitment to infection sites renders them at risk of viral infection, which could affect their effector activity. CD8 T lymphocytes express RIG-I, which detects cytosolic viral RNA and subsequently induces antiviral gene expression. We investigated how Influenza A virus infection and synthetic triphosphorylated double-stranded RNA, a specific RIG-I ligand, influence TCR-dependent effector responses in primary human CD8 T cells. Cells were isolated from healthy donors and either infected with the reassortant virus RG-PR8-Brazil78 (H1N1) or transfected with the synthetic RNA. Proliferation, degranulation, and cytokine production upon anti-CD3/CD28 stimulation were assessed using flow cytometry and intracellular cytokine staining. Type I IFN production and downstream signaling were measured using IFN-I reporter assay and Western blotting. CRISPR/Cas9 gene editing was employed to knock out RIG-I and STAT2 to evaluate their roles in antiviral responses. Influenza A virus infection of CD8 T cells stimulated RIG-I and activated downstream pathways, including TBK1 and NF-κB, resulting in type-I interferon secretion. Transfection of cytotoxic CD8 T lymphocytes with synthetic RIG-I ligands not only stimulated these pathways but also enhanced the proliferation of CD8 T cells in vitro and protected them from influenza A virus infection. In line with a positive effect on CD8 effector function, both influenza A virus infection and RIG-I ligand transfection enhanced CD8 T cell degranulation and cytokine secretion. Conversely, activation of CD8 T lymphocytes via CD3/CD28 crosslinking increased their susceptibility to influenza A virus infection. We demonstrated that RIG-I stimulation by virus infection or RIG-I ligand transfection promotes intrinsic antiviral pathways and enhances CD8 T-cell effector functions and proliferation. This suggests that RIG-I agonists could enhance and prolong the effector function of cytotoxic CD8 T lymphocytes in immunotherapy.

## 1. Introduction

Cytotoxic CD8 T cells are integral immune components that effectively target pathogen-infected or transformed cells [1]. Their main mode of action is the recognition and elimination of abnormal cells presenting specific antigens on HLA class I molecules. This is mediated by direct killing through the release of perforin and granzyme B, as well as the production of pro-inflammatory cytokines such as TNF and IFN-γ [2,3,4], which enhance cytotoxicity and amplify the immune response [5,6]. Owing to the nature of TCR-dependent activation, which requires direct cell contact [7] between CD8+ T cells and infected target cells, there is potential for significant exposure to infectious virions, which may in turn impact their capacity for subsequent responses.

CD8+ T cells have been shown to be susceptible to infection with a number of viruses. For example, in vitro HHV-6 infection of Jurkat T cells and peripheral blood T cells induced the expression of the CD4 protein on mature CD8 T cells, rendering them vulnerable to HIV-1 infection [8,9]. Epstein–Barr virus (EBV), while having tropism for B cells, has also been found to infect other cells, including CD8+ T cells, which is clinically obvious in patients with EBV-positive T-cell lymphoproliferative disease [10]. Similarly, certain strains of EBV-2 readily infect purified CD8 T cells and induce activation, proliferation, and altered cytokine expression [11]. Although human T-lymphotropic virus 1 (HTLV-I) primarily infects CD4 T cells, it has been suggested that CD8 T cells may serve as an additional reservoir for HTLV-I in infected patients [12]. In addition to DNA viruses, negative-sense single-strand RNA viruses such as measles virus (MeV) and influenza A virus (IAV) have also been reported to infect CD8 T cells. The peripheral blood mononuclear cells (PBMCs) of prodromal measles patients were shown to be infected by MeV with specific tropism for CD8+ T cells [13], and in vitro experiments demonstrated that MeV can replicate in T lymphocytes, including CD8+ T cells [14]. In mouse models, lymphocytic choriomeningitis virus (LCMV) can infect CD8 T cells, and influenza A virus (IAV) has also been shown to directly infect CD8 T cells [15]. Consequently, T-cell-intrinsic antiviral mechanisms may significantly impact not only the susceptibility of T cells to viral infection but also their subsequent TCR-dependent responses.

Autonomous antiviral defense mechanisms have been described in immune cells, with a substantial impact on their effector response. The activation of these innate nucleic acid receptors typically triggers a robust antiviral immune response characterized by the secretion of type I IFN (IFN-I), cytokines, and chemokines. Toll-like receptors (TLRs) 3, 7, 8, and 9 sense viral nucleic acids within endosomes, whereas cytosolic RNA and DNA are sensed by retinoic acid-inducible gene I (RIG-I)-like helicases (RLHs) and cyclic GMP-AMP synthase (cGAS), respectively [16,17,18,19]. Many studies have shown that ligand recognition by TLRs expressed on APCs plays a crucial role in T-cell activation [20], but the role of intrinsic T-cell recognition of nucleic acids is less well-described. Direct stimulation of TLR3 expressed on CD8 T cells results in increased IFN-γ production without affecting their cytolytic function or proliferative capacity [21]. Similarly, in vitro analyses demonstrated that direct exposure of human CD8 T cells to TLR7 ligands directly increased the expression of the activation markers CD69 and CD25, along with the production of IFN-γ [22].

Few studies have directly addressed the potential of ligand recognition by T-cell-intrinsic RNA helicases to modulate T-cell function. RIG-I is probably the best described of these helicases and specifically recognizes structural RNA motifs that are a common feature of viral RNA or replicative intermediates that are generated during viral infection [17,23,24,25]. RIG-I signals through the activation of the mitochondrial antiviral signaling protein (MAVS), which activates IkappaB kinases (TBK1/IKKα/IKKβ/IKKε), which in turn activate transcription factors such as nuclear factor kappa B (NF-κB) and IRF3/7 [18]. In macrophages and dendritic cells, this typically results in the secretion of type-I and type-III interferons and cytokines such as IL-27 [26]. The secreted IFN-I then signals through the IFN-α/β receptor complex (IFNAR) to activate Janus kinase 1 (Jak1) and tyrosine kinase 2 (Tyk2), which subsequently phosphorylate the signal transducer and activator of transcription 1 (STAT1) and STAT2 proteins [27]. Phosphorylated STAT2 in association with STAT1 and interferon regulatory factor 9 (IRF-9) forms interferon-stimulated gene factor 3 (ISGF3) [25]. Once translocated to the nucleus, it activates the transcription of a range of antiviral effector genes, including the interferon-induced protein with tetratricopeptide repeats 1 (IFIT1) [18,28]. Synthetic ligands such as short (>/=20 bp) 3p-dsRNAs mimic viral RNA and are potent and specific inducers of the RIG-I signaling pathway [17].

While RIG-I deficiency has been shown to diminish T-cell responses following IAV infection, where deficient mice exhibit lower levels of activated CD8 T cells [29], it is unclear whether this is due to direct effects on RIG-I expressed by CD8 T cells themselves or rather reflects indirect effects such as reduced activation or cytokine production by APCs. This study investigated the impact of intrinsic CD8+ T-cell activation of RIG-I on effector function via either direct infection of CD8+ T cells with IAV or the transfection of pure RIG-I ligands (3p-dsRNAs) into the cytosol of CD8+ T cells. The data show that in vitro IAV infection induced RIG-I and downstream TBK1 and NF-κB pathways, as well as the secretion of type-I IFN in infected cells. Second, although activated CD8 T cells are more susceptible to IAV infection, their effector functions, such as degranulation and the secretion of IFN-γ and TNF, are enhanced by IAV infection. Moreover, targeted activation of RIG-I with 3p-dsRNA drove similar responses, namely, increased release of type-I IFN and increased degranulation and IFN-γ secretion by CD8+ T cells. Importantly, this RIG-I activation provided protection against subsequent IAV infection and enhanced the proliferative capacity of the cells. Overall, these findings demonstrate that RIG-I activation in negatively selected CD8 T cells has the potential to enhance the effector function of these cells and offers a new avenue to improve the outcomes of T-cell therapies.

## 2. Results

### 2.1. Activated CD8 T Cells Are More Susceptible to IAV Infection

**Figure 1:**
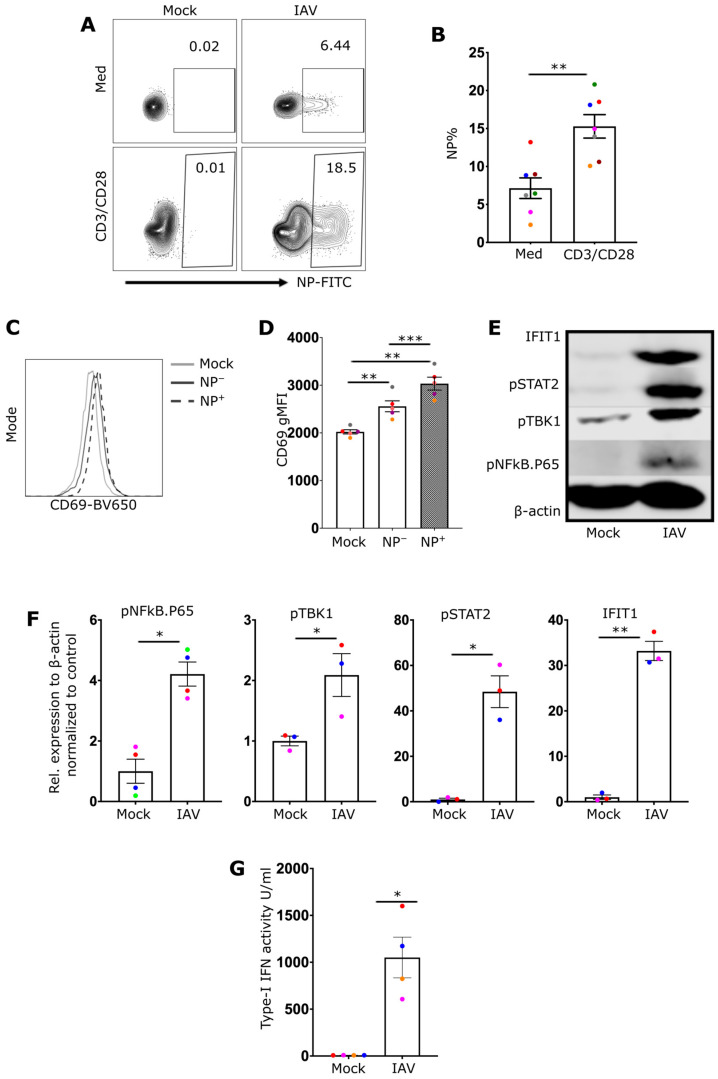
Infection of human primary CD8 T cells leads to the activation of NF-κB, TBK1 and interferon response. (**A**) Representative flow cytometry plots showing the viral nuclear protein (NP) expression in CD8 T cells cultured for 3 days with or without anti-CD3/CD28 antibodies, then treated with either media (mock) or infected with 10 MOI of IAV for 8 h. (**B**) A bar chart summarizing the results for 7 different donors infected with IAV with or without anti-CD3/CD28 antibodies. (**C**) Representative histogram of CD69 expression in mock-treated, NP−, and NP+ cells from the infection condition (*n* = 7). (**D**) Quantification of CD69 gMFI (*n* = 5). (**E**) Western blot image of mock- and IAV-infected CD8 T cells, illustrating different proteins associated with nucleic acid receptor stimulation (pNFkB-P65 and pTBK1) and interferon response (IFIT1 and pSTAT2) (*n* = 3–4). (**F**) Quantification of the proteins illustrated in (**E**). (**G**) A bar chart demonstrating the IFN-I activity detected by TBK1^−/−^ IKKα^−/−^ IKKβ^−/−^ & IKKε^−/−^ THP1 dual reporter cells (*n* = 4). Every donor is represented by a colored dot; bars show mean ± SEM. Paired *t*-test was used for two group comparison and one-way ANOVA followed by Dunnett’s correction for more than two groups (* *p* < 0.05, ** *p* < 0.01, and *** *p* < 0.001).

To assess the impact of exposure to IAV on CD8+ T-cell function, resting or in vitro-activated CD8+ T cells were initially infected with IAV for 8 h, after which the proportion of virally infected cells expressing IAV nuclear protein (NP+) was determined via flow cytometry. The proportion of NP+ cells was significantly greater following anti-CD3/CD28 stimulation (16% vs. 7%) (Figure 1A,B). The differences in these proportions of NP+ cells were not attributable to differences in cell viability, as they were not significantly different between the groups at 25 h post infection (Supplementary Figure S1A). Moreover, although there was clear evidence of infection at 25 h post infection, the absence of newly released infectious virus in the supernatant indicated that the infection was not productive (Supplementary Figure S1B).

### 2.2. IAV Drives RIG-I, NF-κB, and TBK1 Activation and the Type-I IFN Response in CD8+ T Cells

The next objective was to assess whether CD8 T cells undergo activation in response to IAV by measuring CD69 expression, an early indicator of cellular activation. Compared with mock-treated cells, IAV-exposed CD8+ T cells presented slightly increased levels of CD69 expression. Moreover, among IAV-exposed cells, NP+ cells presented higher levels of CD69 expression than did the NP- population (Figure 1C,D).

As IAV infection can activate nucleic acid sensors, potentially driving the production of cytokines, the impact of IAV infection on different pathways downstream of nucleic acid receptors was assessed. Protein immunoblotting revealed that IAV infection induced the phosphorylation of both NF-κB.p65 and TBK1 (Figure 1E,F, Supplementary Figures S5 and S6). Additionally, type-I IFN was detected in the supernatants of infected CD8+ T cells 24 h post infection (Figure 1G). Type-I IFNs can drive an autocrine signaling cascade by binding to IFNARs, which results in STAT2 phosphorylation. Consistent with signaling through IFNARs, immunoblotting revealed STAT2 phosphorylation in IAV-infected but not mock-treated CD8+ T cells (Figure 1E,F). In line with the production and recognition of IFN-I, the expression of IFIT1, an interferon-induced protein, was also strongly induced in IAV-exposed cells (Figure 1E,F). Although the phosphorylation of NF-κB may be driven by exposure to exogenous cytokines, the activation of TBK1 and the release of type-I IFN in IAV-infected CD8+ T cells demonstrate the involvement of upstream nucleic RNA receptors, such as RIG-I, in the activation process.

**Figure 2:**
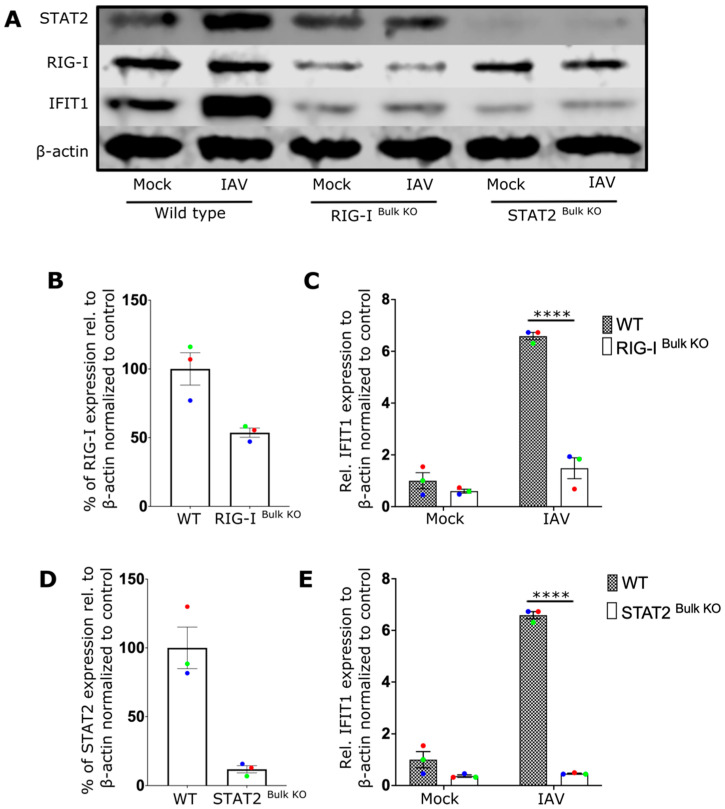
IAV infection triggers antiviral IFN response in CD8 T cells via RIG-I and STAT2 signaling pathways. (**A**) Representative Western blot image of wildtype (WT), RIG-I ^Bulk KO^, and STAT2 ^Bulk KO^ CD8 T cells treated with media (Mock) or infected with influenza A virus (IAV). (**B**,**D**) show the relative expression level of RIG-I and STAT2 respectively in WT and knockout cells. (**C**,**E**) displays the relative expression level of IFIT1 in WT cells and RIG-I ^Bulk KO^ or STAT2 ^Bulk KO^ cells treated as mentioned. Every donor is represented by a colored dot (*n* = 3); bars show mean ± SEM. Two-way ANOVA followed by Bonferroni’s correction for more multiple comparison (**** *p* < 0.0001).

To better understand the role of RIG-I receptors in the CD8 T-cell IFN response during viral infection, CRISPR editing was used to delete RIG-I or STAT2 from primary CD8 T cells. As a key downstream component of IFNAR, STAT2 is crucial for the autocrine amplification loop that enhances T-cell activation. Investigating this could clarify the role of the amplification loop. The efficiency of the knockout was evaluated by analyzing protein expression relative to that of beta-actin. Immunoblot analyses revealed that the protein expression of both RIG-I (Figure 2A,B, Supplementary Figures S7–S9) and STAT2 (Figure 2A,D) was reduced by more than 50% in the CRISPR-edited cells. Moreover, the expression of IFIT1 following IAV infection was essentially abrogated in both RIG-I- and STAT2-knockout cells, demonstrating the importance of both RIG-I signaling and STAT2 signaling in the interferon response through autocrine activation of IFNAR (Figure 2C,E).

### 2.3. IAV Infection Increases the Effector Function of CD8+ T Cells

**Figure 3:**
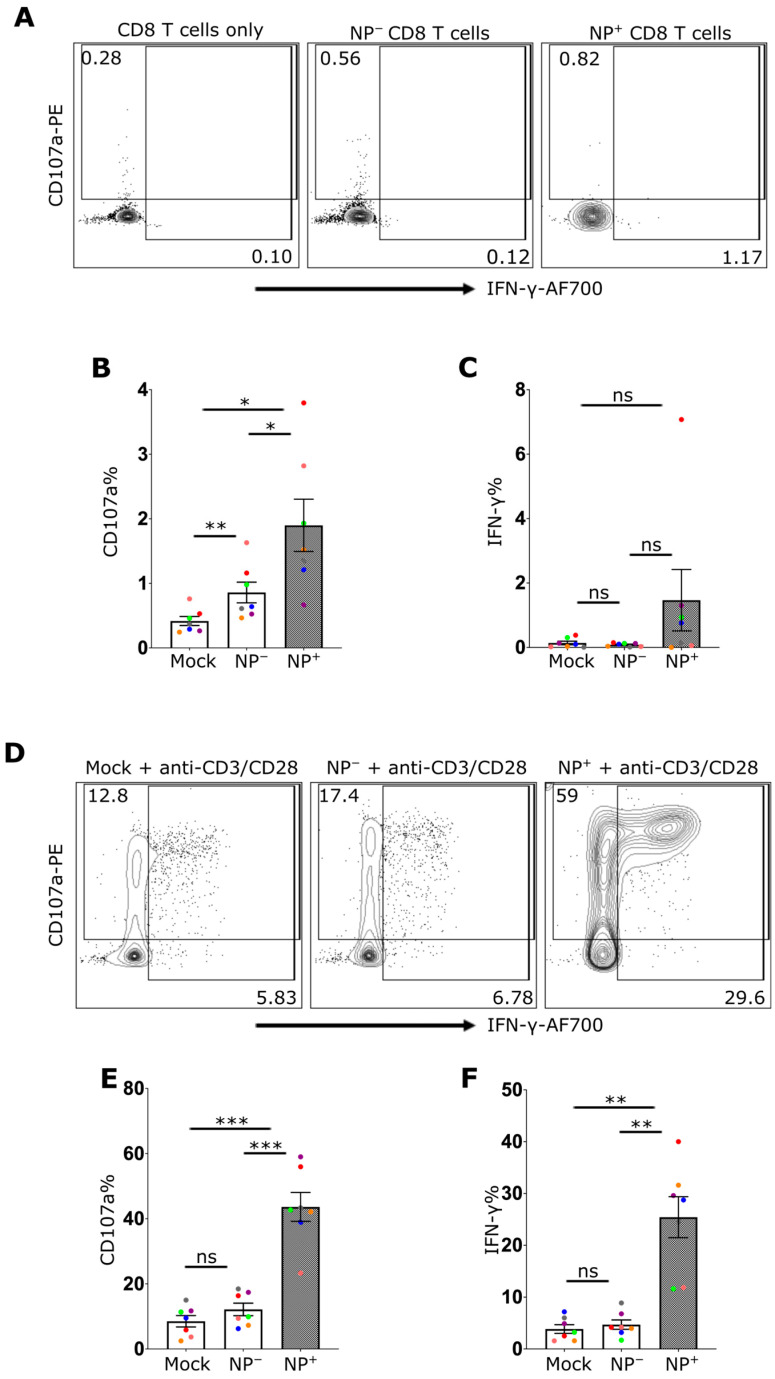
Enhanced Effector Function in CD8 T Cells upon IAV Infection. Flow cytometry analysis of CD8 T cells 5 h post-infection with or without activation. A and D display the representative dot plots of CD107a and IFN-γ expression in CD8 T cells without anti-CD3/CD28 antibody activation (**A**) or with anti-CD3/CD28 antibody activation (**D**). Cells were treated with media control (Mock) or infected with IAV, gated based on the expression of viral nuclear protein: IAV-exposed cells (NP−) or infected cells (NP+). (**B**,**C**) show the frequencies of CD107a+ (**B**) or IFN-γ+ (**C**) CD8 T cells without activation, while (**E**,**F**) show the frequencies with antibody activation. Every donor is represented by a colored dot (*n* = 7); bars show mean ± SEM. Repeated measure one-way ANOVA followed by Dunnett’s correction for more than two groups (ns = not significant, * *p* < 0.05, ** *p* < 0.01, and *** *p* < 0.001).

The observation that IAV infection of CD8 T cells activated nucleic acid-sensing pathways, resulting in both the activation of NF-κB and the secretion of IFN-I, suggested that IAV infection also has the potential to modulate effector functions. To assess this more formally, CD8 T cells were cultured in plates coated with or without anti-CD3 and anti-CD28 antibodies for three days and then infected with IAV for 5 hr. After infection, degranulation was assessed by staining for CD107a, and cytokine production was assessed by staining for intracellular IFN-γ. As expected, in the absence of the stimulating mAbs, neither mock-treated nor IAV-infected CD8+ T cells exhibited notable degranulation or IFN-γ production (Figure 3A–C). Following stimulation, both the degranulation and IFN-γ responses of IAV-exposed but uninfected (NP−) CD8+ T cells were similar to those of mock-treated controls (Figure 3D–F). However, the responses of NP+ CD8+ T cells were significantly elevated (Figure 3E,F), suggesting an intrinsic impact of IAV in enhancing CD8+ T effector function beyond simple exposure to secreted IFN-I (Figure 3).

### 2.4. 3p-dsRNAs Trigger the Activation of the TBK1 and NF-κB Pathways in CD8+ T Cells, Resulting in the Induction of Type I Interferons

**Figure 4:**
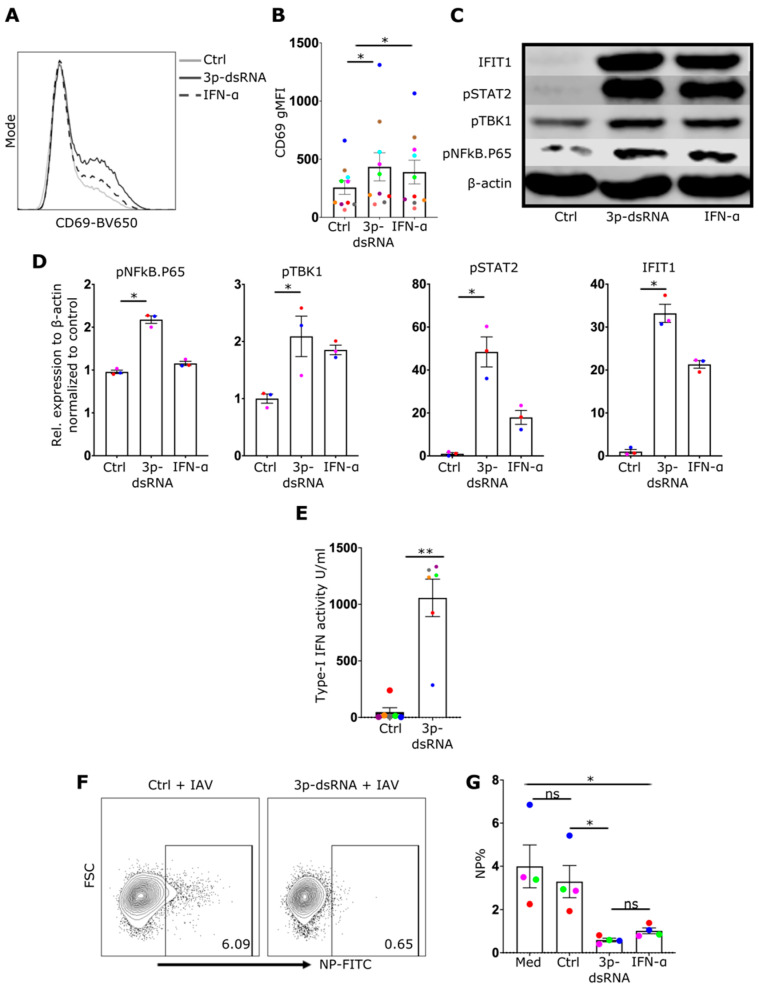
RIG-I stimulation in CD8 T cells stimulates the same pathways as in IAV infection and inhibits subsequent IAV infection. (**A**) An illustrative histogram displaying CD69 expression in CD8 T cells that were treated with control (Ctrl), RIG-I ligands (3p-dsRNA) or IFN-α. (**B**) Quantification of CD69 gMFI (*n* = 10). (**C**) Western blot image showing proteins from CD8 T cells that were treated with the same conditions as mentioned in (**A**). (**D**) Bar charts illustrating the relative levels of listed proteins to β-actin in CD8 T cells (*n* = 3). (**E**) Bar chart shows the measurement of IFN-I activity using THP1 reporter cells (*n* = 6). (**F**) Flow cytometry plots showing CD8 T cells treated as mentioned and then infected with IAV for 8 h. (**G**) Bar chart showing the frequencies of infected cells (NP+) for CD8 T cells treated as labelled (*n* = 4). Every donor is represented by a colored dot; bars show mean ± SEM. Paired *t*-test was used for two group comparison and repeated measures one-way ANOVA followed by Dunnett’s correction for more than two groups (ns = not significant, * *p* < 0.05, ** *p* < 0.01).

While the data clearly showed that IAV infection could activate RIG-I to drive IFN-I production, the enhanced effector responses could be attributed to broader effects of IAV infection on CD8+ T-cell function. Consequently, to better target RIG-I, a specific in vitro-generated RNA ligand, 3p-dsRNA, or control RNA (which does not activate RIG-I), was introduced into CD8 T cells. Flow cytometry using FAM-labeled 3p-dsRNA confirmed the successful delivery of the RIG-I ligand into CD8 T cells, as indicated by the presence of FAM+ cells (Supplementary Figures S2A and S10). Similar to the response observed with IAV, the introduction of 3p-dsRNA induced the upregulation of the activation marker CD69 (Figure 4A,B), phosphorylation of NF-κB-p65 and TBK1 (Figure 4C,D, Supplementary Figure S11), type-I IFN secretion (Figure 4E), and phosphorylation of STAT2 downstream of IFN-I receptors (Figure 4D), ultimately leading to the induction of the interferon-stimulated protein IFIT1 (Figure 4C,D).

In addition to RIG-I stimulation enhancing the activation of CD8 T cells, pretreatment with RIG-I ligand (or IFN-I), in contrast to negative control RNA, protected NP+ CD8 T cells from IAV infection (Figure 4F,G).

**Figure 5:**
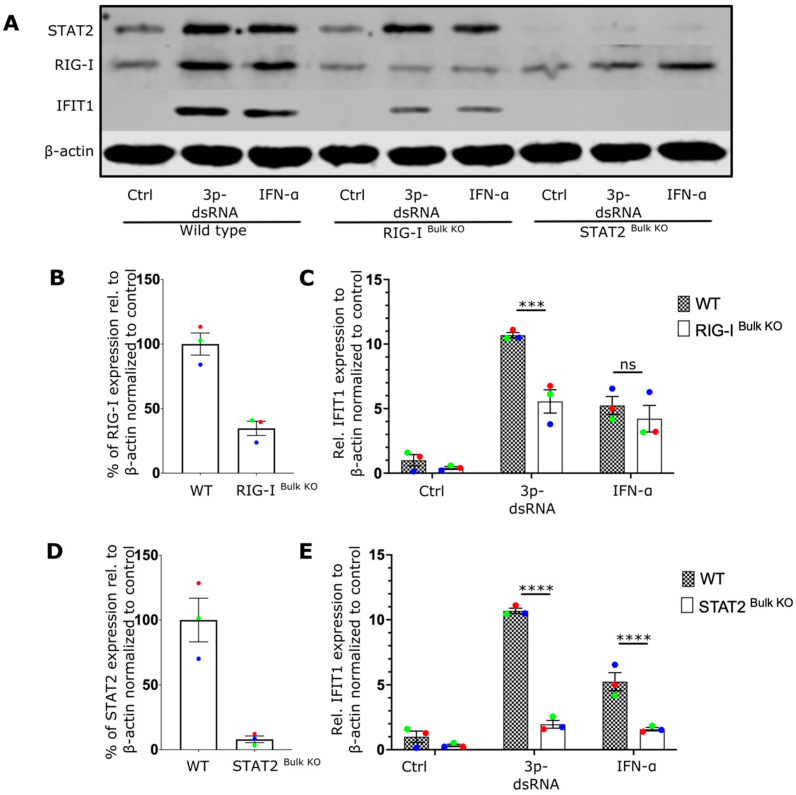
RIG-I ligands trigger interferon response in CD8 T cells via RIG-I and STAT2 pathways. (**A**) Representative Western blot displays the expression of IFIT1, RIG-I, and STAT2 proteins in CRISPR/Cas9 genetically edited CD8 T cells treated as described. The cells included wildtype (WT), RIG-I ^Bulk KO^, and STAT2 ^Bulk KO^ cells. (**B**,**D**) Bar chart showing the relative expression levels of RIG-I and STAT2 respectively. (**C**,**E**) The bar charts display the relative expression levels of IFIT1 in RIG-I ^Bulk KO^ cells and STAT2 ^Bulk KO^ respectively. Every donor is represented by a colored dot (*n* = 3); bars show mean ± SEM. Two-way ANOVA followed by Bonferroni’s correction for multiple comparisons (ns = not significant, *** *p* < 0.001, and **** *p* < 0.0001).

Similar to IAV infection, the transfection of 3p-dsRNA resulted in reduced induction of IFIT1 by 5-fold in RIG-I- and by 6-fold in STAT2-deficient CD8 T cells compared with unmodified T cells (Figure 5, Supplementary Figures S12–S14), further confirming that RIG-I stimulation is upstream of the NF-κB and TBK1 pathways, which leads to type-I IFN secretion in CD8+ T cells. Furthermore, the addition of neutralizing antibodies to block IFNAR2 resulted in a reduction in STAT2 phosphorylation and IFIT1 protein expression, confirming that both effects depend on the autocrine/paracrine effects of secreted IFN-I (Supplementary Figure S2B).

### 2.5. RIG-I Ligands Increase the Function of CD8 T Cells

**Figure 6:**
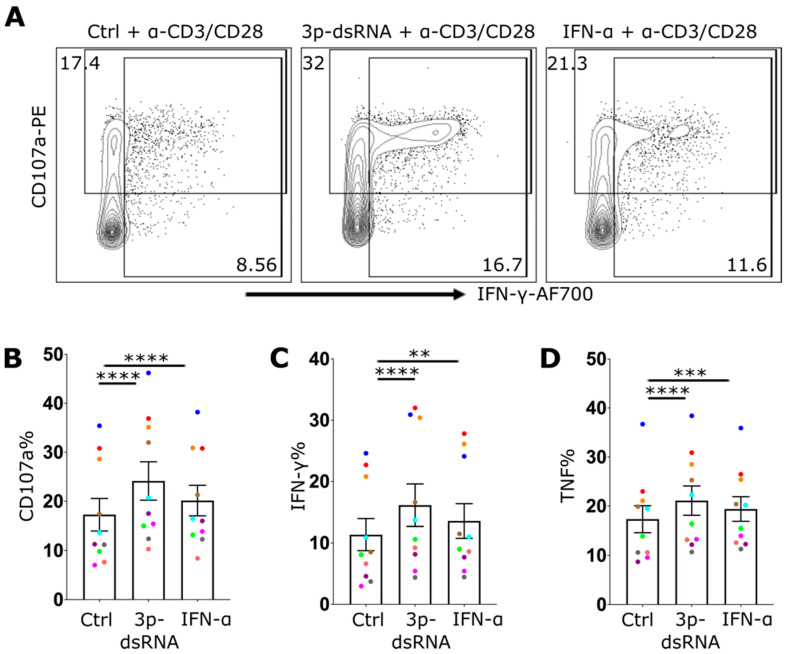
RIG-I stimulation in CD8 T cells enhances their effector function. (**A**) Flow cytometry plot showing CD8 T cells treated with control RNA (Ctrl), 3p-dsRNA or IFN-α overnight followed by anti-CD3/CD28 activation for 4 hrs. The plot displays the expression of CD107a and IFN-γ. (**B**–**D**) Bar charts illustrating the frequencies of CD107a+ (**B**), IFN-γ+ (**C**) and TNF+ (**D**) CD8 T cells after antibody-mediated activation. Every donor is represented by a colored dot (*n* = 10); bars show mean ± SEM. Repeated measures one-way ANOVA followed by Dunnett’s correction for more than two groups (** *p* < 0.01, *** *p* < 0.001, and **** *p* < 0.0001).

To determine whether the enhanced effector response of infected CD8 T cells was directly attributable to RIG-I pathway CD8 T-cell effector function, CD8 T cells were treated with control RNA, 3p-dsRNA or IFN-α, and their response to antibody activation was evaluated. In line with the observations during IAV infection, a significant increase in degranulation (Figure 6A,B), as well as in the production of IFN-γ (Figure 6A,C) and TNF (Figure 6D), was observed after exposure to 3p-dsRNA. On the other hand, compared with the control, IFN-α led to enhanced effector function but to a lesser extent than RIG-I ligands did. Additionally, enhanced effector function in response to antibody activation was observed in sorted CD8 T cells (Supplementary Figure S3), ruling out the potential effects of contaminating myeloid cells. These findings suggest that the increased activation of CD8 T cells following exposure to 3p-dsRNA was due to intrinsic activation of the RIG-I pathway within CD8 T cells themselves. In summary, CD8 T effector function is significantly enhanced by IAV and 3p-dsRNA via an RIG-I-dependent mechanism.

### 2.6. RIG-I Activation Enhances the Proliferation of CD8+ T Cells

**Figure 7:**
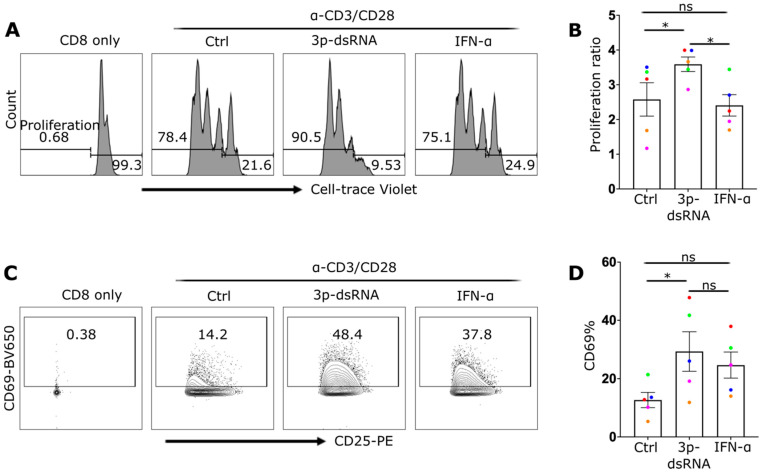
RIG-I ligands enhance CD8 T cell activation and proliferation. (**A**) Histograms showing CD8 T cells with conditions as labelled. The leftmost peak represents the control group with no stimulation or proliferation. The other peaks represent cells stimulated overnight with control RNA (Ctrl), 3p-dsRNA, or IFN-α, followed by a 3-day culture in CD3/CD28 coated plates. The histogram demonstrates the proliferation tracking dye (Cell-trace Violet), with the presence of multiple peaks indicative of dye dilution with cell division. (**B**) The proliferation ratio of each condition calculated by dividing the total cell count of the anti-CD3/CD28-treated cells by cells incubated with no anti-CD3/CD28. (**C**) Flow cytometry plots for the same conditions as in (**A**), displaying CD69 on the Y axis and CD25 on the X axis. (**D**) presents the CD69 frequencies. Every donor is represented by a colored dot (*n* = 5), bars show mean ± SEM. Repeated measures one-way ANOVA followed by Dunnett’s correction for more than two groups (ns = not significant, * *p* < 0.05).

RIG-I stimulation enhanced TCR-mediated responses, which suggested that it may influence not only activation and the effector response but also proliferation. We compared the proliferation of RIG-I-stimulated CD8 T cells with 3p-ssRNA (negative control) and IFN-α, which are known to activate these cells [30]. Compared with those stimulated with the negative control or IFN-α, the proliferation of CD8+ T cells stimulated with RIG-I was greater. This was shown by the higher percentage of proliferating cells (cell trace violet low) (Figure 7A) and the higher proliferation ratio, which was determined on the basis of the cell counts (Figure 7B). While CD25 expression remained similar across all conditions, the expression of the activation marker CD69 was significantly increased in RIG-I- and IFN-α-stimulated CD8 T cells (Figure 7C,D), with RIG-I ligands showing the highest expression levels. These findings indicate that RIG-I stimulation may have a beneficial effect on the prolonged activation of CD8+ T cells, leading to increased cell expansion.

## 3. Discussion

During the process of targeting infected cells, immune cells such as CD8 T cells often encounter infectious virions, especially at synaptic junctions, potentially increasing their vulnerability to infection [31]. The direct effect that this process can have on CD8 T-cell effector function has not been thoroughly explored. Previous studies have highlighted the significant indirect impact of ligand recognition by nucleic acid receptors on CD8+ T-cell responses [29,32,33]. This was shown to occur through increased activation of antigen-presenting cells such as dendritic cells, leading to increased secretion of immunomodulator cytokines and increased expression of co-stimulating molecules [34]. There is, however, evidence that intrinsic T-cell activation of nucleic acid receptors can significantly alter their function. For example, TLR3 receptor stimulation in murine CD8+ T cells resulted in increased IFN-γ production without affecting their cytolytic or proliferative activity [21]. Similarly, in vitro studies have shown that TLR7 ligands can directly amplify the activation and cytokine production of human CD8+ T cells [22]. In contrast, stimulation of the DNA receptor cGAS/STING pathway induces IFN-I but negatively affects T-cell functions by increasing cell death and decreasing proliferation [35].

RIG-I has been proposed to have therapeutic potential by enhancing the effector function of CD8+ T cells. The administration of RIG-I ligands into mice synergistically complemented the outcome of checkpoint inhibition, leading to the activation and expansion of antigen-specific CD8 T cells ex vivo and enhancing their antitumor response in vivo [36]. However, the role of intrinsic RIG-I activation in CD8+ T cells has not been addressed.

Although T cells are not the primary viral targets, evidence in both human and mouse systems suggests their susceptibility to viral infections, including IAV infection [8,9,13,15,37,38,39]. Several studies have shown that viruses such as IAV and HTLV-I preferentially infect activated human lymphocytes compared with resting cells [40,41]. Increased susceptibility of activated T cells may be attributed to distinct glycosylation profiles, including higher expression of sialyl glycans that facilitate viral entry [42]. In addition, elevated metabolic activity in activated cells may support viral replication and enhance viral protein expression [43]. In contrast, resting T cells may exhibit stronger intrinsic antiviral responses, limiting infection efficiency. This concept is supported by findings in the HIV field, where resting CD4+ T cells are less susceptible to infection compared with activated CD4 T cells [44]. Consistent with these observations, our data demonstrate that activated CD8+ T cells are more susceptible to IAV infection than resting cells. Alternatively, it is possible that naïve CD8+ T cells undergo rapid cell death upon infection, resulting in a lower proportion of NP^+^ cells detected by flow cytometry. Further investigation is required to clarify the mechanisms underlying the increased proportion of NP+ CD8+ T cells following activation.

Our results indicate that the most distinct effect of IAV infection is via activation of the RIG-I pathway. This not only enhances CD69 expression but also activates the downstream TBK1 and NF-κB pathways and IFN-I secretion. Furthermore, we used primary CRISPR/Cas9-edited CD8 T cells to analyze the associated signaling pathways. The induction of the IFN-I response in CD8 T cells following either IAV infection or 3p-dsRNA transfection is dependent on RIG-I. Although IFIT1 is known to be directly regulated by RIG-I receptor signaling through IRF3 [45], we observed that deleting STAT2 downstream of IFNAR nearly abolished the induction of IFIT1 expression in CD8 T cells, indicating a crucial role of the autocrine IFN feedback/amplification loop via IFNAR in CD8 T cells for the full induction of the antiviral response.

Functionally, RIG-I activation has a costimulatory effect on TCR-mediated activation. This was mainly observed for infected cells (NP+) but not for noninfected bystander cells, demonstrating a strong dependence on the intracellular RIG-I signal transduction rather than being dependent on secreted IFN-I from these cultures. Additionally, treatment with IFN-α had a lesser effect on CD8 T-cell effector function than RIG-I ligands did, further suggesting that the synergy between RIG-I and TCR-mediated signaling depends on more RIG-I-induced signaling molecules and genes than solely IFN-I. These results highlight the potential of RIG-I receptor signaling to amplify CD8 T-cell function, suggesting its potential as a therapeutic target for enhancing therapies based on CD8 T cells. Importantly, RIG-I signaling not only protects CD8+ T cells from infection and enhances their effector function but also strongly promotes the proliferation of CD8+ T cells. This was unexpected, since the stimulation of the cGAS/STING pathway, which shares parts of cytosolic signaling with the RIG-I pathway, inhibits T-cell growth and therefore impedes T-cell effector function [35].

NF-κB signaling may be the key pathway responsible for the activating effects of RIG-I, as NF-κB is activated by both RIG-I [46] and TCR- [47] signaling but not by IFNAR.

This study sheds light on the impact of inherent RIG-I pathway activation on cellular responses during viral infections, particularly those involving IAV. These findings demonstrate that RIG-I activation can enhance TCR-dependent activation of CD8 T cells, affecting processes such as degranulation, proliferation, and cytokine release and providing defense against subsequent IAV infections. Additionally, we show that CD8 T cells are capable of secreting IFN-I via direct RIG-I ligand transfection or IAV infection. These findings reveal an expanded role for these cells in combating viral infections and immune challenges. In addition to their direct antiviral and anticancer effects, the ability of CD8 T cells to secrete IFN-I enables them to stimulate other immune cells and alert noninfected cells to the viral threat, thereby enhancing the overall immune response.

Taken together, these results suggest the potential therapeutic use of RIG-I activation to enhance protective immune responses against viral pathogens. More extensive research is needed to comprehensively investigate the therapeutic implications and real-life effects of RIG-I activation on antiviral immunity. A deeper understanding of how viral infections interact with the adaptive immune response, particularly CD8+ T-cell-mediated immunity, could lead to innovative immunotherapeutic approaches. These approaches aim to effectively combat viral or other microbial infections and enhance the immune response against tumors.

One of the applications of T-cell therapy is CAR-T-cell therapy. CAR-T cells have revolutionized the treatment of certain cancers but still face limitations, including lengthy manufacturing times, suboptimal persistence during and after ex vivo cultivation, and reduced efficacy against solid tumors [48]. RIG-I stimulation, which enhances T-cell proliferation, offers a promising approach to overcome these challenges. By increasing T-cell expansion, RIG-I stimulation could shorten the manufacturing process, improve the persistence of CAR-T cells during cultivation and after reinfusion, and enhance their effectiveness against solid tumors. This could make CAR-T-cell therapy more accessible and effective, addressing some of the current hurdles in its application.

Recent studies have analyzed CD8 T cell function in tumor killing and found enhanced anti-tumor responses of RIG-I-deficient CD8 T cells [33,49]. Notably, this RIG-I-mediated regulatory mechanism occurs in the absence of RIG-I RNA ligands and functions through HSP90 sequestration, which interferes with STAT5 signaling and inhibits the AKT/glycolysis signaling pathway. These findings suggest the signaling-independent role of upregulated apo-RIG-I in the absence of viral infection or RIG-I stimulation [33,49]. In contrast, our data show that RIG-I stimulation enhances CD8 T cell function, suggesting that RIG-I acts as a context-dependent regulator of CD8 T cell responses, promoting or restraining their activity depending on whether activating stimuli, such as viral infection, are present. Since the last decade, the cytosolic dsDNA sensor cGAS and the signaling hub STING downstream of cGAS, on the one hand, and RIG-I-like helicases, on the other hand, have been in antitumor therapy, as reviewed previously [50,51]. Our data suggest that RIG-I ligands have an important advantage over cGAS/STING agonists, as they are able to directly enhance CD8 T-cell effector function and proliferation.

## 4. Materials and Methods

Mononuclear cells from the peripheral blood (PBMCs) of healthy individuals were collected through a density gradient centrifugation process after receiving written consent and approval from the relevant institutional review board. CD8+ T cells were purified from isolated PBMCs via the EasySep™ Human CD8+ T-Cell Isolation Kit (Stemcell Technologies, Vancouver, BC, Canada, #17953) through negative selection. These isolated cells were then cultured in RPMI 1640 medium (Thermo Fisher Scientific, Langerwehe, Germany, #11875093) containing 10% fetal bovine serum (FBS) (Thermo Fisher Scientific, Langerwehe, Germany, #10437028), 100 U/mL penicillin, 100 μg/mL streptomycin (Thermo Fisher Scientific, Langerwehe, Germany, #15140122), and 100 U/mL human IL-2 (Miltenyi Biotec, Bergisch Gladbach, Germany, #130-097-743) at 37 °C in a humidified atmosphere of 5% CO_2_. To measure the activity of type-I interferon in the supernatant of infected and transfected CD8 T cells, a reporter monocytic human cell line called THP1 dual knockouts (TBK1^−/−^ IKKα^−/−^ IKKβ^−/−^ IKKε^−/−^) was used. This cell line lacks expression of TBK1, IKKα, IKKβ, and IKKε but not interferon signaling and was cultured at 37 °C in a humidified atmosphere of 5% CO_2_ in RPMI 1640 medium supplemented with 10% fetal bovine serum, 100 U/mL penicillin, and 100 μg/mL streptomycin.

### 4.1. Proliferation of CD8+ T Cells

U-shaped 96-well plates were coated overnight at 4 °C with 100 µL of 2 mg/mL anti-CD3 (BD Bioscience, San Jose, CA, USA, #555336) and 2 mg/mL anti-CD28 antibodies (BD Bioscience, #555725) in PBS. As a control, the cells were cultured in wells that contained only PBS overnight. On the following day, the purified CD8 T cells were stained with CellTrace™ Violet Cell Proliferation dye (1 µg/mL) for 15 min, washed and resuspended in fresh RPMI media supplemented with 10% FBS, 100 U/mL penicillin, 100 μg/mL streptomycin, 100 U/mL human IL-2, and 20 ng/mL IL-15. After that, the antibody cocktails were removed from the coated wells, and 1 × 10^5^ purified CD8+ T cells were added to each well. The cells were then cultured for 3 days at 37 °C in a humidified atmosphere of 5% CO_2_. When cell counting was needed, 20,000 beads were added before flow cytometry acquisition.

### 4.2. IAV Infection and RIG-I Stimulation of CD8+ T Cells

CD8 T cells were exposed to the reassorted influenza A virus (IAV) strains from PR8 and A/Brazil/11/1978: RG-PR8-Brazil78 HA and NA (H1N1) at a multiplicity of infection of 10 in serum-free medium. After one hour, the cells were washed twice with PBS and then incubated for various durations depending on the experiment. RIG-I ligands were generated via a Transcript Aid T7 in vitro transcription kit (Thermo Fisher Scientific, Langerwehe, Germany, #K0441) with annealed DNA oligonucleotides serving as dsDNA templates, as previously described [52] (sequence: TTGTAATACGACTCACTATAGGGACGCTGACCCAGAAGATCTAGAAATAGTAGATCTTCTGGGTCAGCGTCCC). A single-stranded 3p-RNA was generated from the dsDNA template (sequence: CGCGCGTAATACGACTCACTATAGGGAGCGCAGACGCGAGCGCGGCACGGCCGCCAAGGCGAGAC) and used as a negative control for RIG-I receptor activation. Lipofectamine 2000 (Thermo Fisher Scientific, Langerwehe, Germany, #11668019) was used to transfect RIG-I ligands. Then, 5 µg/mL RNA and 2.5 µL/mL Lipofectamine 2000 were mixed together and allowed to react for 15 min to form a complex. After that, the mixture was added to 10^6^ NK cells/mL for stimulation. In some experiments, recombinant IFN-α2a (1000 U/mL) was used to stimulate the cells (Miltenyi Biotec, Bergisch Gladbach, Germany, #130-108-984).

### 4.3. Western Blot

After harvesting, equal numbers of cells were centrifuged at 500× *g* for 5 min and washed with PBS. The cells were then lysed with 1x Laemmle buffer, which included PhosStop (Roche, Mannheim, Germany, #4906837001) and protease inhibitor (Roche, #4693116001). The lysate was vortexed and incubated at 95 °C for 5–7 min in a thermomixer and shaken at 600 rpm to denature the bound proteins. The lysate was loaded onto a 10% SDS–PAGE gel for electrophoresis until the desired separation was achieved. The proteins were then transferred to nitrocellulose membranes and individually stained via a sequential staining approach with primary antibodies. The primary antibodies used were anti-β-actin mouse mAb (LI-COR Bioscience, Bad Homburg, Germany, #926-42212), anti-IFIT1 rabbit mAb (Cell Signaling, Danvers, MA, USA, #14769), anti-phospho-p65 rabbit mAb (Cell Signaling, #3033), anti-phospho-TBK1 rabbit mAb (Cell Signaling, #5483), anti-RIG-I rabbit mAb (Cell Signaling, Danvers, MA, USA, #3743), anti-STAT2 rabbit mAb (Cell Signaling, Danvers, MA, USA, #72604) and anti-phospho-STAT2 rabbit mAb (Cell Signaling, Danvers, MA, USA, #88410). After staining, the proteins were imaged via an Odyssey imaging system (LI-COR Biosciences). Image Studio Lite software (version 5.0) was used to quantify the relative expression levels of the target protein by normalizing the signal intensity of the target protein to the signal intensity of β-actin.

### 4.4. Flow Cytometry and Degranulation Assay

The cells were rinsed with FACS buffer consisting of 2% FBS and 0.5 µM EDTA in PBS and then incubated at 4 °C with anti-hCD3 BV-510 (BD Bioscience, #563109), anti-hCD8 APC (Miltenyi Biotec, Bergisch Gladbach, Germany, #130-110-679) or FITC (BD Bioscience, #561948), anti-hCD69-BV650 (BD Bioscience, #563835), anti-hCD107a-PE (BD Bioscience, #555801) antibodies, anti-hCD25-PE (BD Bioscience, #555432), and Fixable Viability Dye eFluor™ 780 (Thermo Fisher Scientific, Langerwehe, Germany, #65-0865-14) for 30 min. To perform the degranulation assay, 50,000–100,000 CD8+ T cells were stimulated with anti-hCD3 (2 µg/mL) and anti-hCD28 (2 µg/mL) antibodies for 4 h at 37 °C. During the entire assay, Golgi Stop (BD Bioscience, #554724), Golgi Plug (BD Bioscience, #555029), and anti-hCD107a-PE antibodies were added to the media. For intracellular protein staining, the cells were fixed, permeabilized with eBioscience™ Foxp3/Transcription Factor Staining Buffer Set (Thermo Fisher Scientific, Langerwehe, Germany, #00-5523-00), and then incubated with anti-hIFN-γ-AF700 (BD Bioscience, #557995), anti-hTNF-PECy7 (BD Bioscience, #560678), and NP-FITC (Abcam, Cambridge, UK, #ab210526) for 30 min at 4 °C. Gating was performed for CD8 T cells on the basis of the expression of CD3 and CD8. CD8+ cells were defined as CD3+ CD8+ cells, and the purity was determined to be greater than 90% for every donor (Supplementary Figure S4).

### 4.5. IFN-I Reporter Assay

The type-I IFN reporter activity was determined by collecting cell-free supernatants 16–20 h after infection or stimulation. One hundred microliters of the supernatant were then added to medium-free THP1-dual TBK1^−/−^ IKKα^−/−^ IKKβ^−/−^ IKKε^−/−^ cells and incubated for 24 h. To measure luciferase activity, 30 µL of the supernatant was mixed with 30 µL of a water solution of coelenterazine (1 µg/mL) in a white 96-well F-bottom plate, and the activity was measured immediately via an EnVision 2104 multilabel reader device (PerkinElmer, Waltham, MA, USA).

### 4.6. CRISPR Editing of Primary CD8+ T Cells

We used predesigned crRNAs to modify genes in primary human CD8 T cells via the CRISPR/Cas9 system. To target the RIG-I gene (also known as DDX58), we used two crRNAs aimed at the negative strand (GGATTATATCCGGAAGACCC) and the positive strand (GATCAGAAATGATATCGGTT). Moreover, we employed a predesigned crRNA that directed the Cas9 endonuclease enzyme to cut the positive strand of STAT2 (AAGTACTGTCGAATGTCCAC) specifically. Electroporation was carried out via the P3 Primary Cell 4D-Nucleofector X Kit S (Lonza, Cologne, Germany, #V4XP-3032) and the 4D Nucleofector system (Lonza, Cologne, Germany) with the EH-115 program. We used up to 1.5 × 10^6^ primary human CD8 T cells per reaction to ensure the maximum viability and uptake of the CRISPR/Cas9 mixture. The CRISPR/Cas9 mixture was prepared by combining 2 µL of 100 µM crRNA for each crRNA, equal amounts of tracrRNA, 1.7 µL of Cas9 enzyme, and 1 µL of 100 µM enhancer. The mixture was adjusted to 25 µL per reaction with electroporation buffer (ratio of 16.65 µL of P3 media to 3.65 µL of supplement). After electroporation, the cells were incubated for 3 days before stimulation and infection.

### 4.7. Statistical Analysis

The statistical calculations were executed via GraphPad Prism 9, and each donor was distinguished by a single dot in all figures, with various colors used to distinguish between donors. The bars represent the mean ± standard error of the mean (SEM) across all donors. Paired *t* tests were employed to determine the significance of differences between two groups, whereas repeated measures one-way ANOVA followed by Dunnett’s correction was used for more than two groups. For multiple comparisons, two-way ANOVA followed by Bonferroni correction was used. Statistical significance is denoted by asterisks as follows: * *p* < 0.05, ** *p* < 0.01, *** *p* < 0.001, and **** *p* < 0.0001.

## 5. Conclusions

In summary, our study demonstrates that intrinsic activation of the RIG-I pathway in human CD8 T cells enhances their antiviral effector functions—promoting proliferation, cytokine production, degranulation, and protection against viral infection. This signaling acts in synergy with TCR-mediated activation and operates independently of type I interferon effects alone, underscoring the potential of RIG-I as a promising target for immunotherapeutic intervention.

These findings highlight the broader therapeutic potential of RIG-I stimulation to strengthen protective immune responses against viral pathogens and improve T-cell-based therapies. Future studies are needed to further explore the clinical utility, safety, and long-term impact of RIG-I agonists. A deeper understanding of how viral infections interact with adaptive immune mechanisms, particularly CD8+ T-cell-mediated responses, could improve the development of novel immunotherapeutic strategies.

One such application is in CAR-T-cell therapy, which has transformed cancer treatment but remains limited by lengthy manufacturing, reduced persistence, and suboptimal efficacy in solid tumors. Enhancing CD8 T-cell proliferation through RIG-I stimulation could help overcome these hurdles by accelerating CAR-T-cell expansion, improving their survival during and after cultivation, and boosting their antitumor activity. Harnessing RIG-I signaling may therefore offer a powerful approach to amplify both antiviral immunity and cancer immunotherapy outcomes.

## Data Availability

Data is contained within the article or Supplementary Material.

## Supplementary Materials

The following supporting information can be downloaded at: https://www.mdpi.com/article/10.3390/ijms27073058/s1.
