# Supplementary material for: RIG-I Stimulation Enhances the Effector Function and Proliferation of Primary Human CD8+ T Cells
Source: Int J Mol Sci. 2026 Mar 27;27(7):3058. doi: 10.3390/ijms27073058 (PMC13073767; doi:10.3390/ijms27073058)
Supplement: Supplementary file 1 [file ijms-27-03058-s001.zip › ijms-4166532-supplementary.pdf]

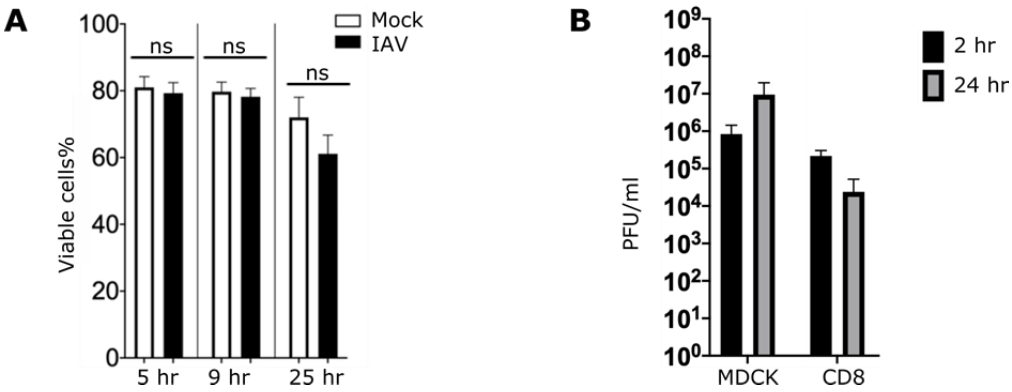

Figure S1. Infection outcomes of IAV in CD8 T cell. Two bar charts showing the results of experiments investigating the effects of influenza A virus (IAV) infection on CD8 T cells. **(A)** a bar chart representing the viability of CD8 T cells mock-infected or infected with IAV at different time points (5, 9, and 25 hrs post-infection). **(B)** display the viral titer (PFU/ml) measured in cell-free supernatants collected from CD8 T cells and MDCK cells positive control at 2 and 24 hrs post-infection (n= 5, mean ± SEM). Two-way ANOVA followed by Bonferroni's correction for multiple comparisons (ns= not significant).

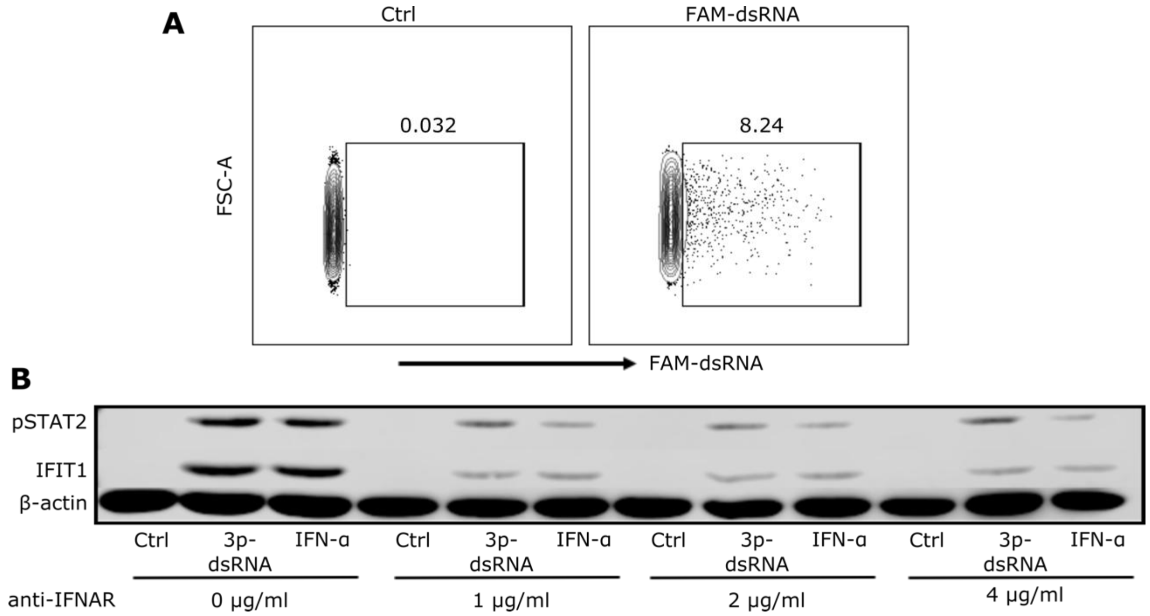

Figure S2. 3p-dsRNA induces type-I IFN secretion and stimulates IFIT1 in an IFNAR dependent mechanism **(A)** Flowcytometry plot showing CD8 T cells treated with unlabeled 3p-ssRNA or FAM-labeled 3p-dsRNA (FAM-dsRNA) **(B)** Western blot image for CD8 T cells either pre-treated without anti-IFNARα antibodies or with 1, 2, or 4 µg/ml for one hr then control RNA (Ctrl), 3pdsRNA, or IFN-α were added. Phospho STAT2 and IFIT1 proteins were used to detect the activation of IFNAR.

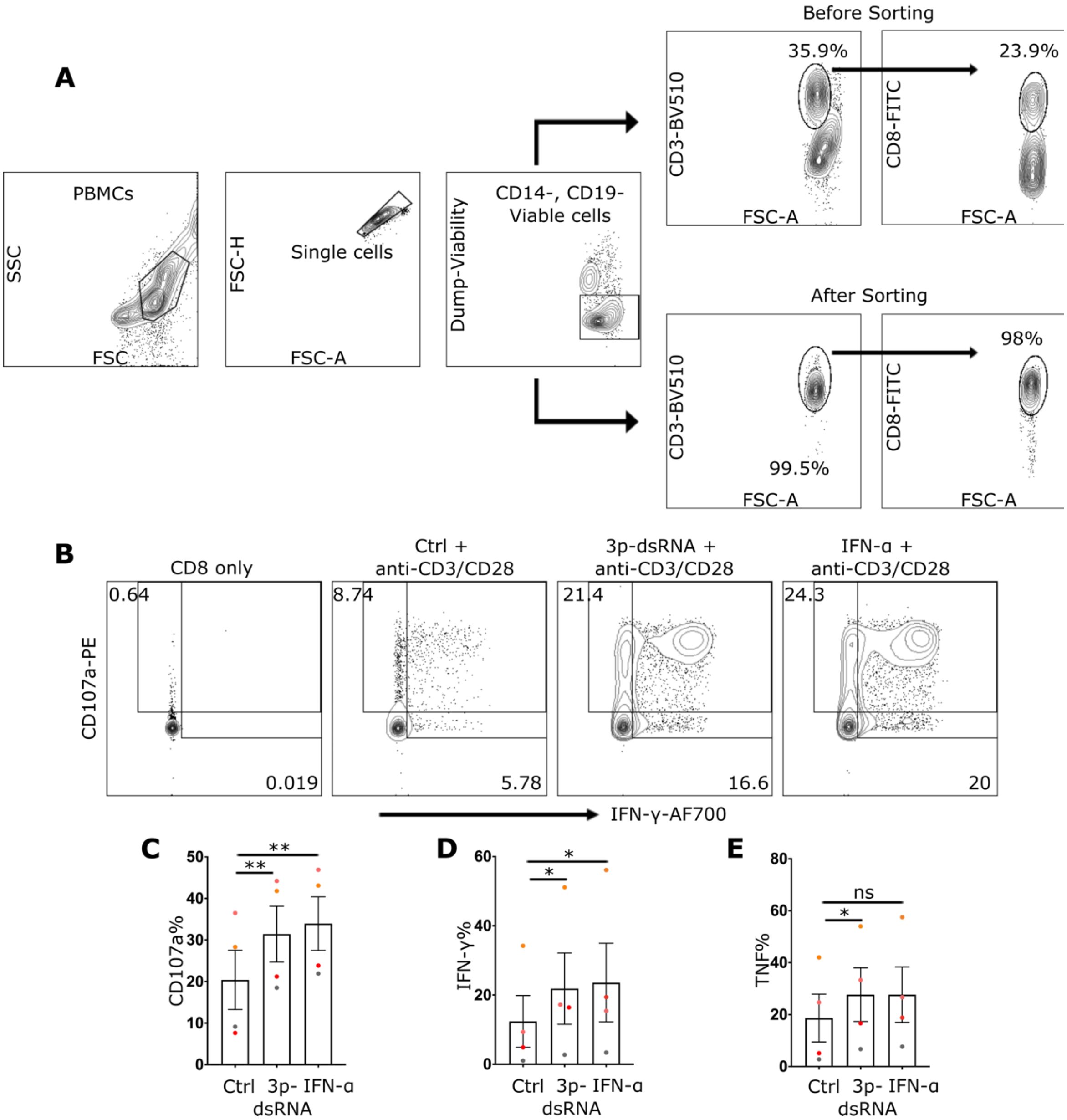

Figure. S3. Sorted CD8 T cells reproduced the results from using purified CD8 T cells (A) Representative flowcytometry plots showing sorting strategy and the purity of CD8 T cells before and after sorting. **(B)** Illustrative flowcytometry plot for sorted CD8 T cells treated with media only, or Ctrl RNA, 3p-dsRNA or IFN-α then stimulated with anti-CD3/CD28 antibodies. The plots demonstrate CD107a against IFN-γ or TNF against CD69 respectively. **(C-E)** quantification of the previously mentioned activation markers. Every donor is represented by a colored dot, bars show mean ± SEM. Repeated measures one-way ANOVA followed by Dunnett's correction for more than two groups (ns= not significant, $*p < 0.05$, and $**p < 0.01$).

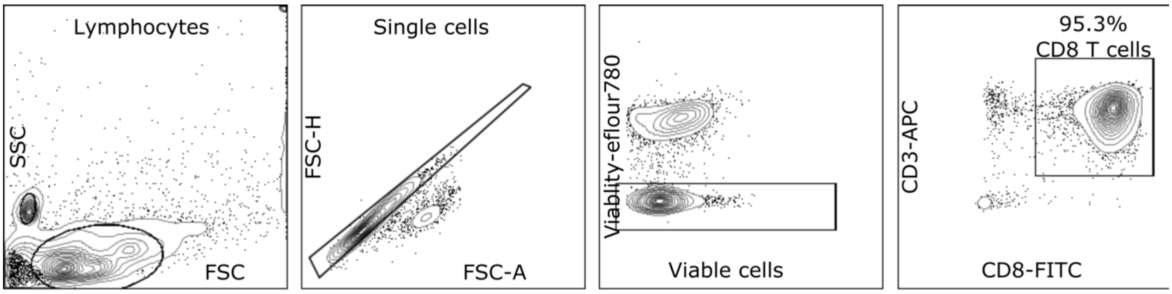

Figure S4. Gating strategy and CD8 T purity Representative flow cytometry plots of peripheral blood mononuclear cells (PBMCs) obtained from one of the donors. The lymphocyte population was initially

gated based on their side and forward scatter characteristics. We then used negative gating to exclude dead cells, and CD8 T cells were identified as cells expressing both CD3 and CD8 markers.

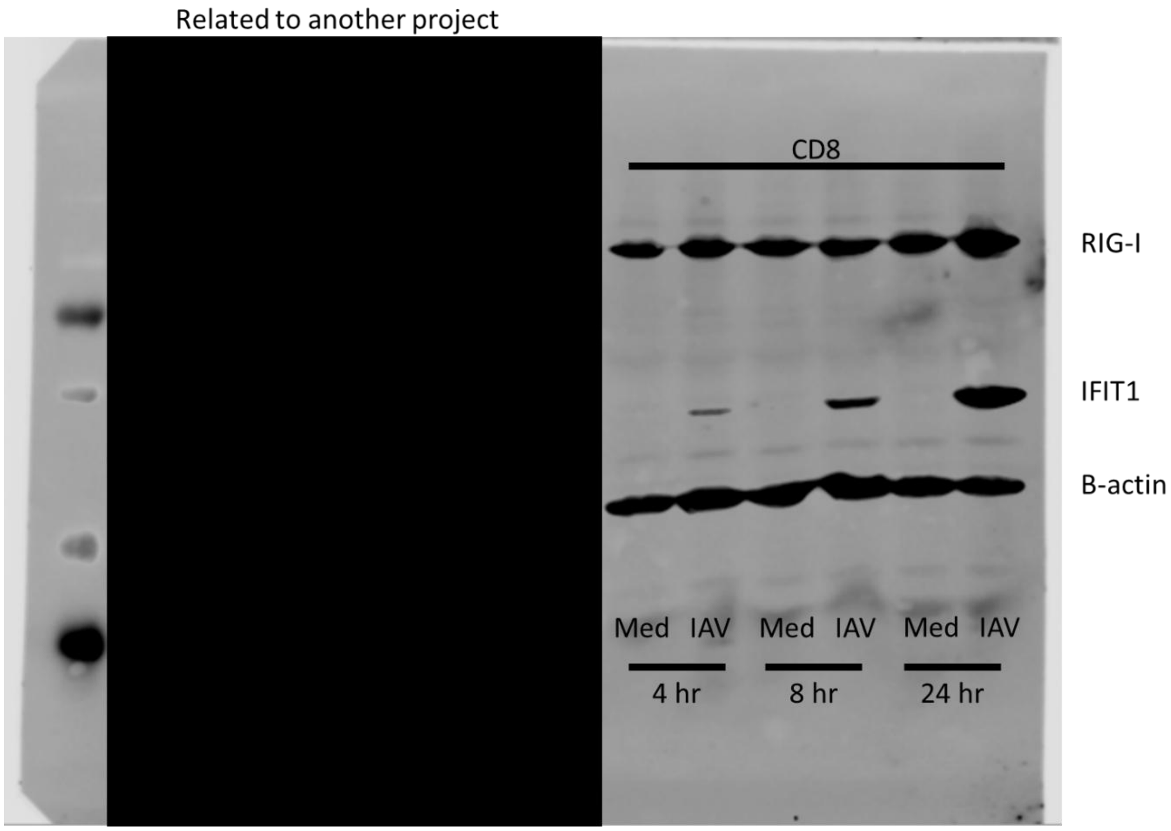

Figure S5. Full image of Western blot membrane showing RIG-I, IFIT1 and B-actin expression in IAV infected CD8 T cells at different time points.

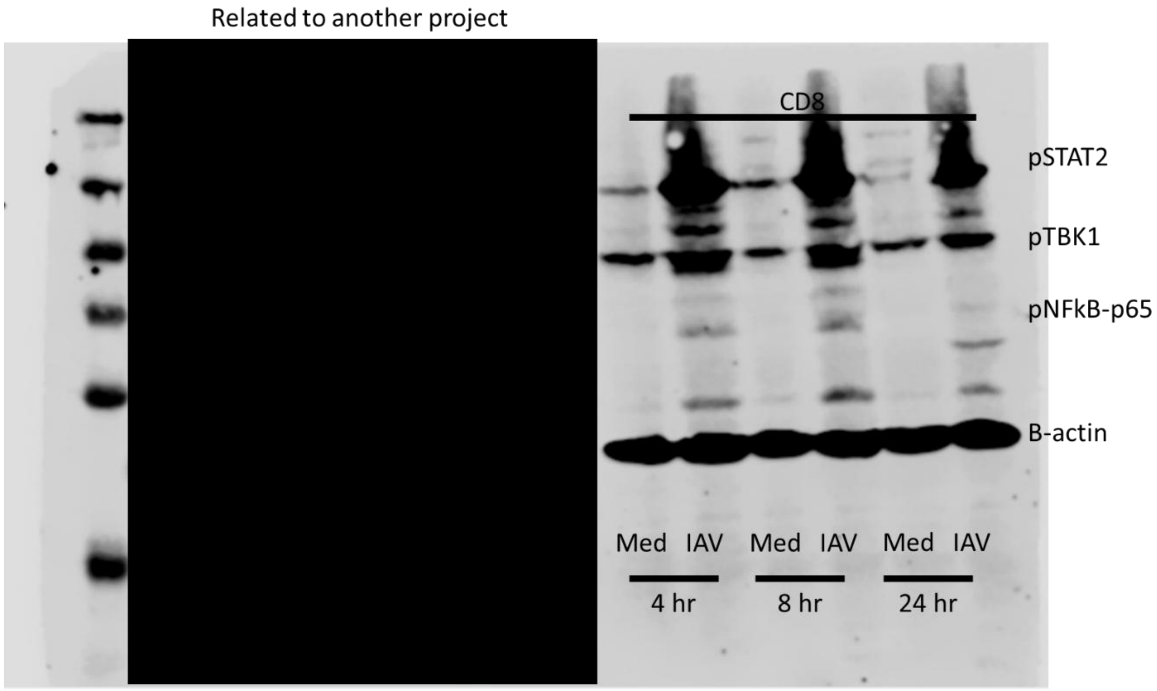

Figure S6. Full image of Western blot membrane showing pSTAT2, pTBK1, pNFkB-p65 and B-actin expression in IAV infected CD8 T cells at different time points

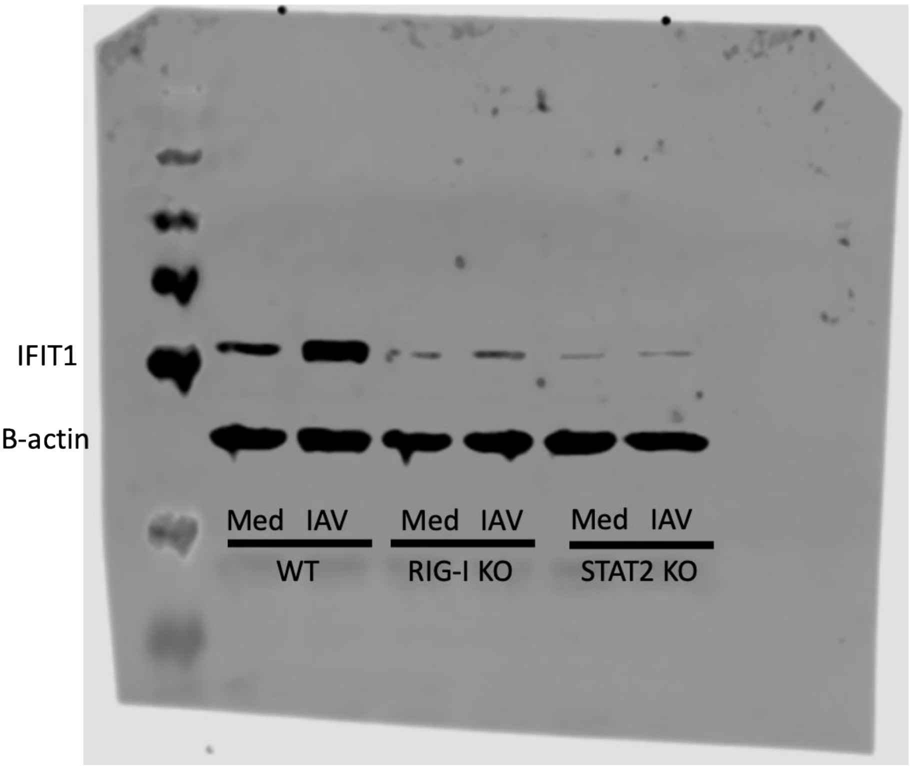

Figure S7. Full image of Western blot membrane showing IFIT1 and B-actin expression in IAV infected WT, RIG-I KO and STAT2 KO CD8 T cells

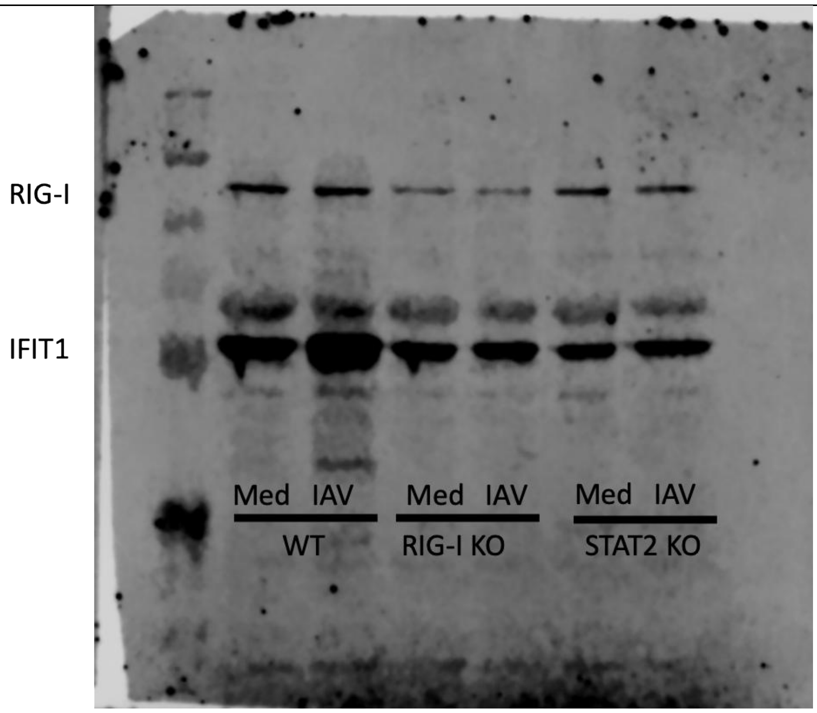

Figure S8. Full image of Western blot membrane showing RIG-I, IFIT1 and B-actin expression in IAV infected WT, RIG-I KO and STAT2 KO CD8 T cells

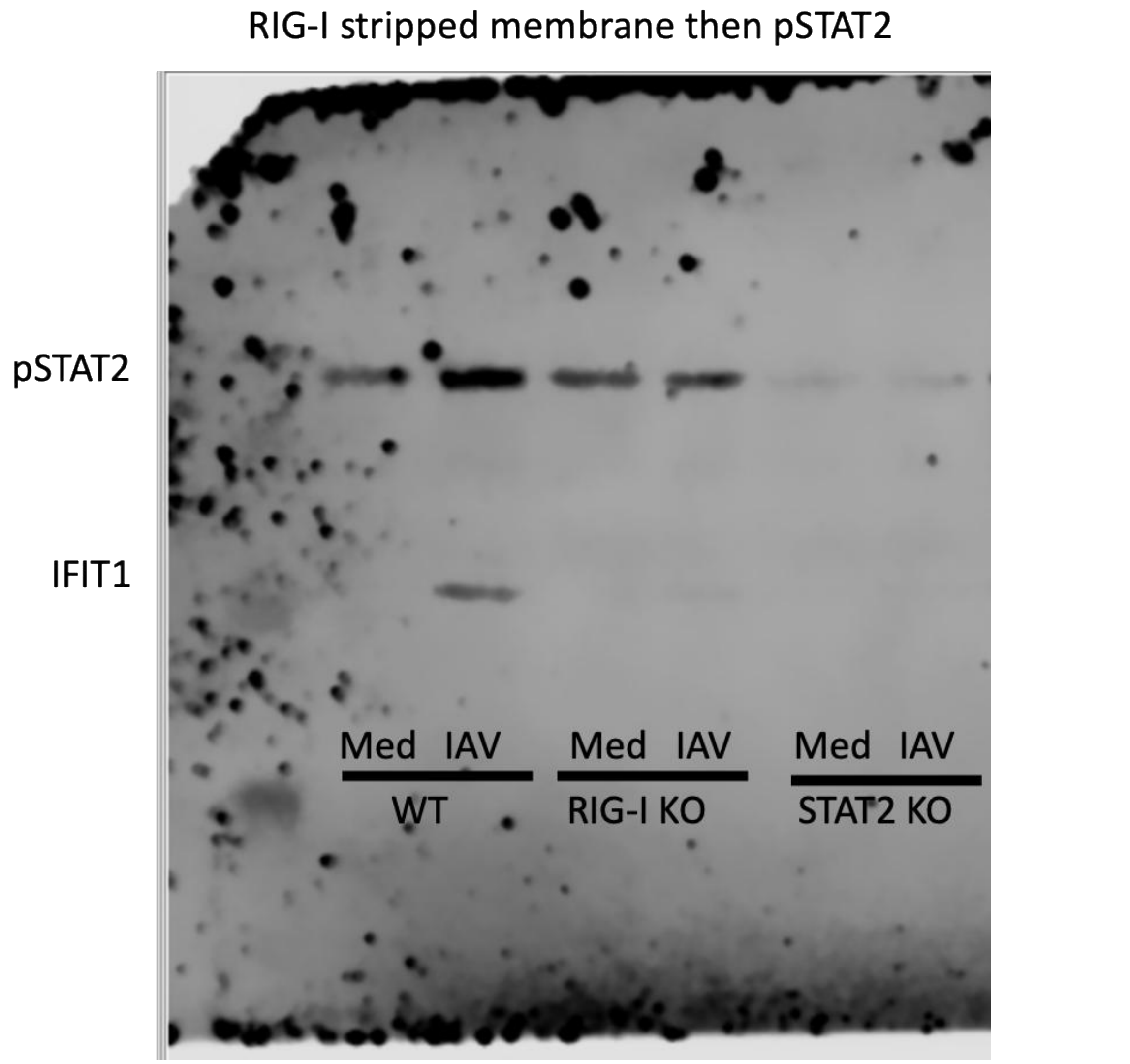

Figure. S9. Full image of Western blot membrane showing STAT2 expression after stripping the previous staining in Supp. Fig. 7 & 8 in IAV infected WT, RIG-I KO and STAT2 KO CD8 T cells

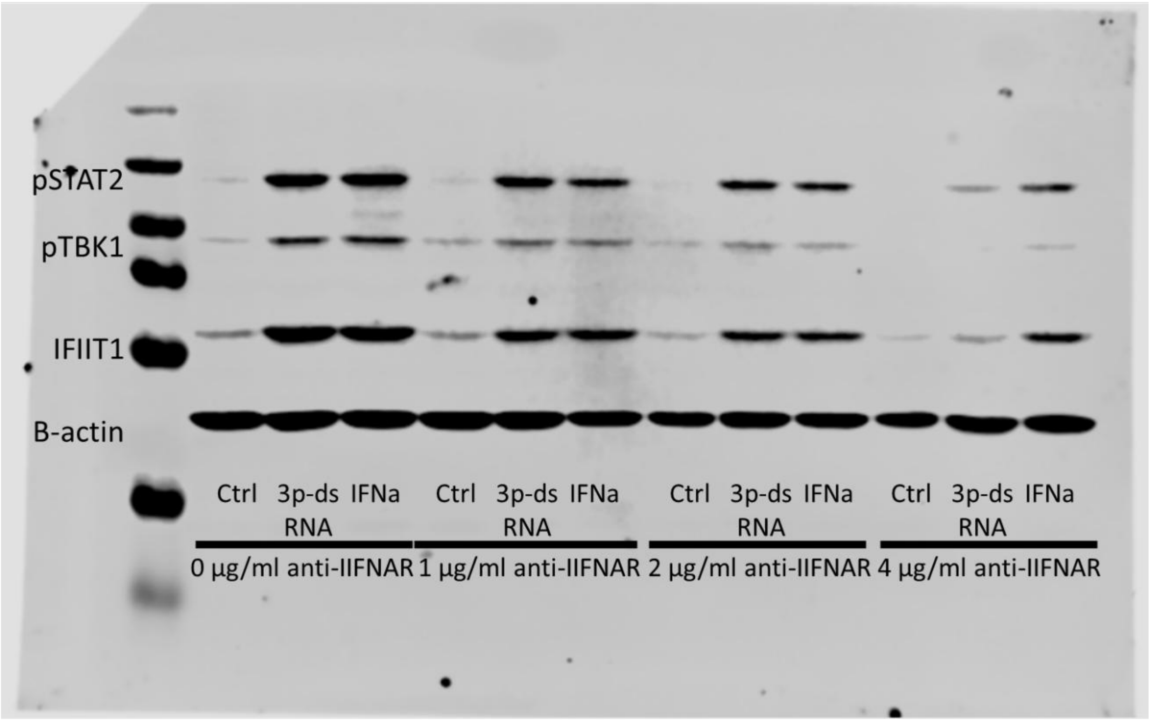

Figure S10. Full image of Western blot membrane showing pSTAT2, pTBK1, IFIT1 and B-actin expression in CD8 T cells treated with different concentrations of anti-IFNAR antibodies incubated with control, RIG-I ligands or IFNa.

Same previous membrane with contrast and brightness adjusted to show differences in pNFkB.P65

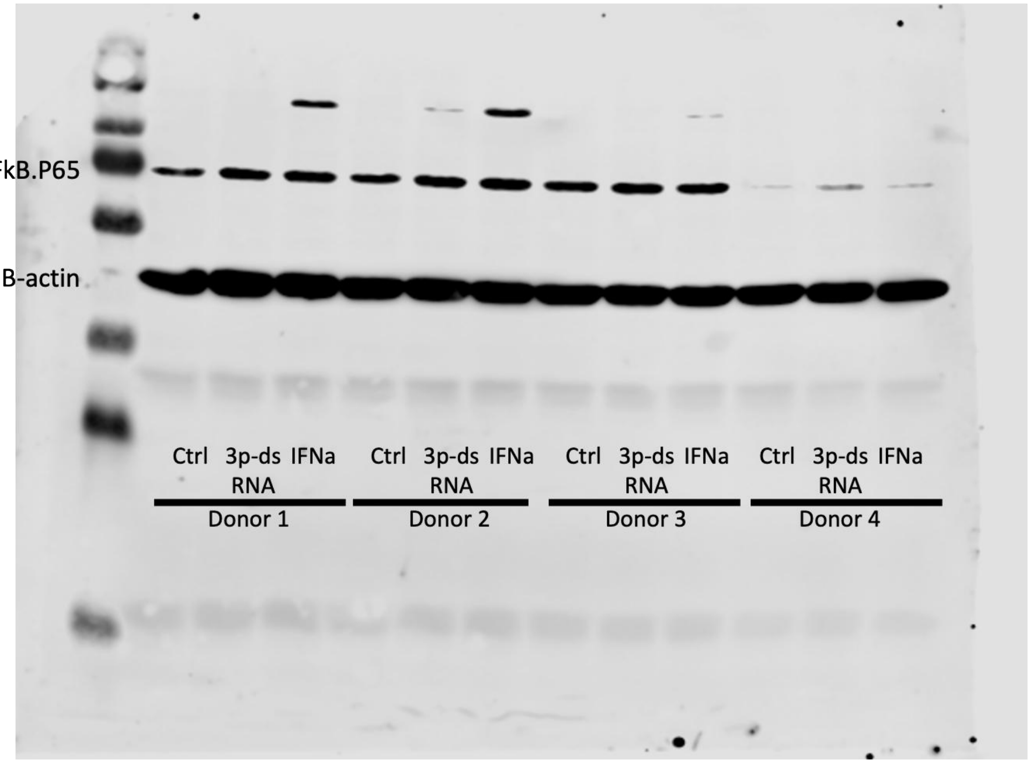

Figure S11. Full image of same Western blot membrane shown in supp. Fig. 11 and sequentially stained with anti-pP65 antibodies but brightness and contrast are adjusted to show the difference in pP65 bands among CD8 T cells from 4 different donors treated with control, RIG-I ligands or IFNa.

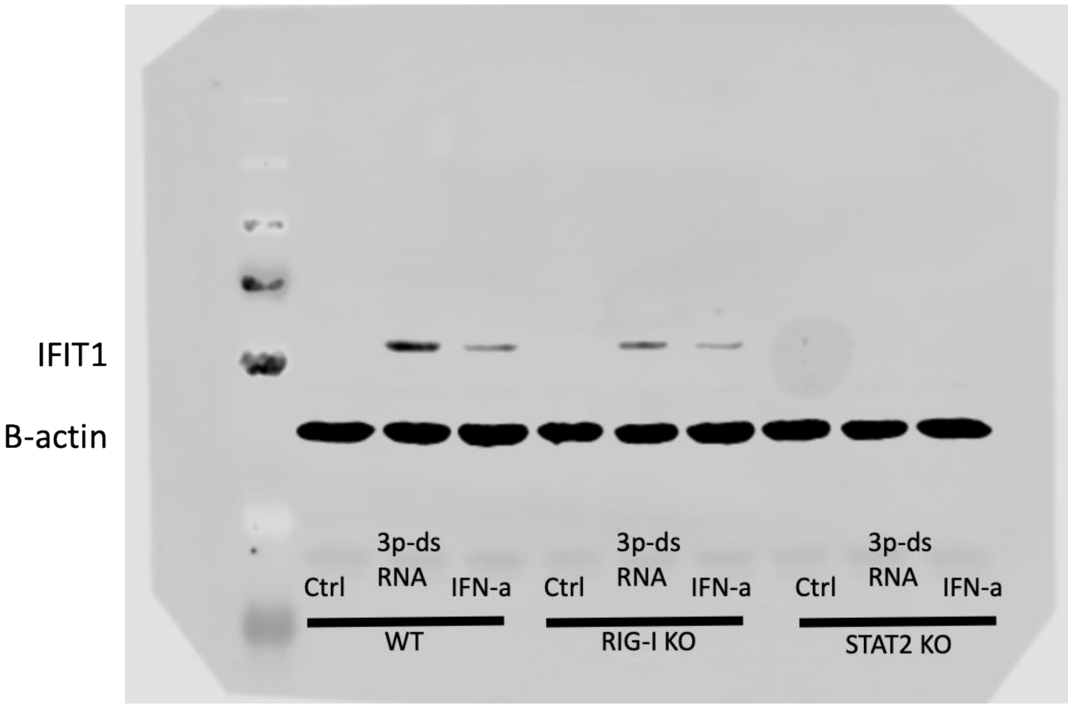

Figure S12. Full image of Western blot membrane showing IFIT1 and B-actin expression in WT, RIG-I KO and STAT2 KO CD8 T cells treated with control, RIG-I ligands or IFNa.

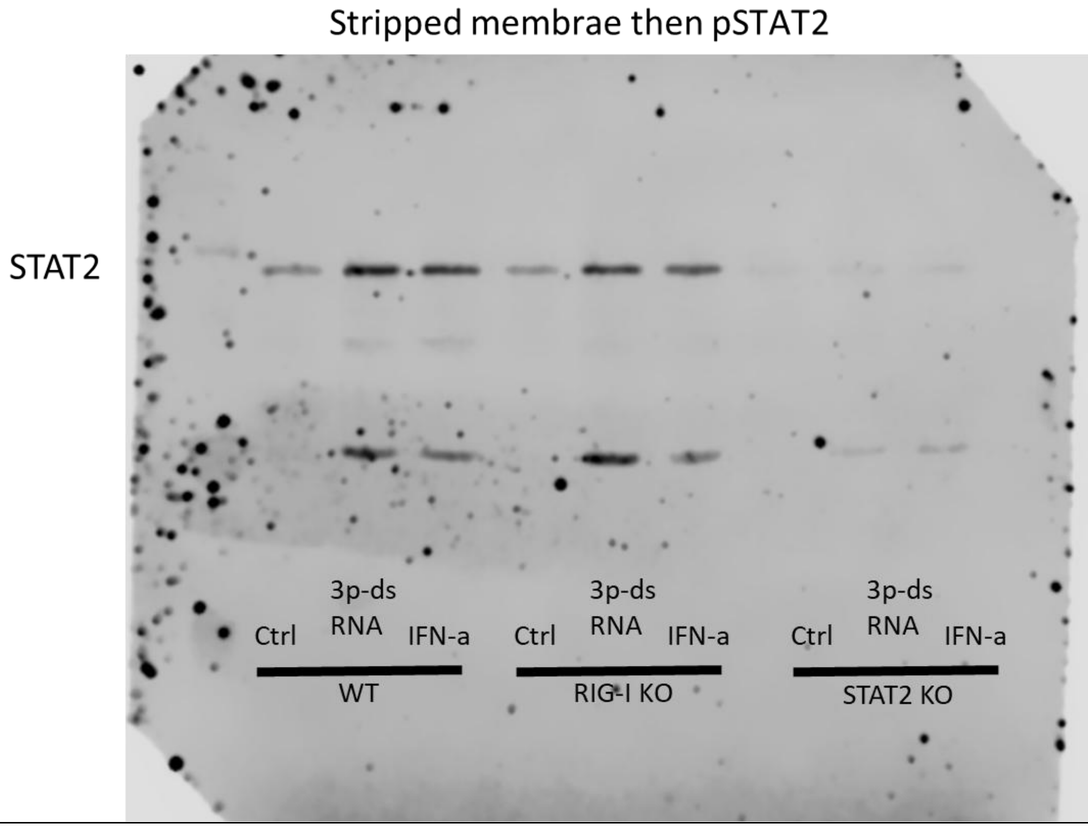

Figure S13. Full image of same Western blot membrane in supp. Fig. 13 stained with anti-STAT2 antibodies after stripping the membrane showing STAT2 expression in WT, RIG-I KO and STAT2 KO CD8 T cells treated with control, RIG-I ligands or IFNa.

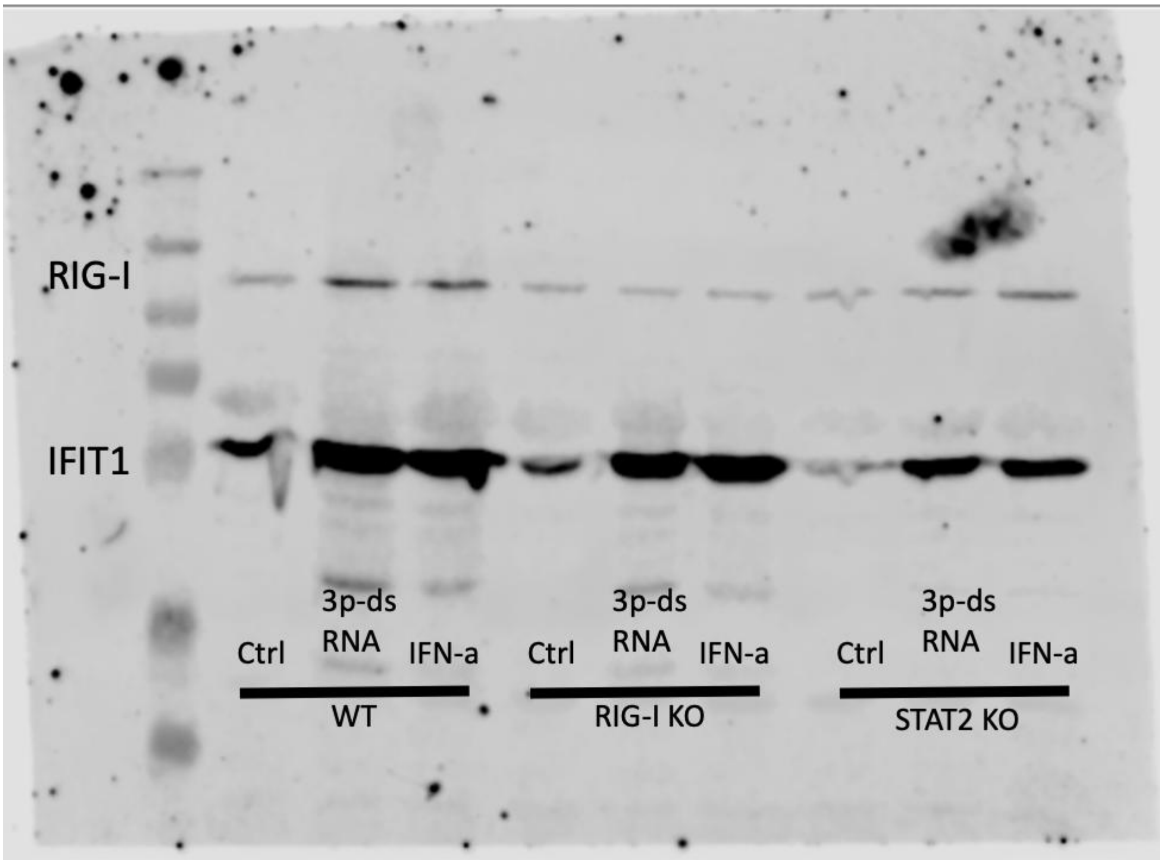

Figure S14. Full image of Western blot membrane loaded with the same cell lysate used in supp. Fig. 13 showing RIG-I and IFIT1 expression in WT, RIG-I KO and STAT2 KO CD8 T cells treated with control, RIG-I ligands or IFNa.
